# Antimicrobial *O*-Alkyl Derivatives of Naringenin and Their Oximes Against Multidrug-Resistant Bacteria

**DOI:** 10.3390/molecules25163642

**Published:** 2020-08-10

**Authors:** Anna Duda-Madej, Joanna Kozłowska, Paweł Krzyżek, Mirosław Anioł, Alicja Seniuk, Katarzyna Jermakow, Ewa Dworniczek

**Affiliations:** 1 Department of Microbiology, Faculty of Medicine, Wroclaw Medical University, Chałubińskiego 4, 50-368 Wrocław, Poland; anna.duda-madej@umed.wroc.pl (A.D.-M.); krojcerpawel@gmail.com (P.K.); alicja.seniuk@umed.wroc.pl (A.S.); katarzyna.jermakow@umed.wroc.pl (K.J.); 2 Department of Chemistry, Faculty of Biotechnology and Food Science, Wrocław University of Environmental and Life Sciences, Norwida 25, 50-375 Wrocław, Poland; miroslaw.aniol@upwr.edu.pl

**Keywords:** naringenin, *O*-alkyl derivative of naringenin, oxime, multidrug-resistant pathogens

## Abstract

New antimicrobial agents are needed to address infections caused by multidrug-resistant bacteria. Here, we are reporting novel *O*-alkyl derivatives of naringenin and their oximes, including novel compounds with a naringenin core and *O*-hexyl chains, showing activity against clinical strains of clarithromycin-resistant *Helicobacter pylori*, vancomycin-resistant *Enterococcus faecalis*, methicillin-resistant *Staphylococcus aureus*, and beta-lactam-resistant *Acinetobacter baumannii* and *Klebsiella pneumoniae.* The minimum inhibitory concentrations (MICs), which provide a quantitative measure of antimicrobial activity, were in the low microgram range for the selected compounds. Checkerboard assays for the most active compounds in combination with antibiotics revealed interactions that varied from synergistic to neutral.

## 1. Introduction

Nowadays, we are facing a growing problem of limitations in the effective treatment of infectious diseases. Infections caused by multidrug-resistant (MDR) bacteria are responsible for significant morbidity and mortality in the healthcare environment. The danger, the level of which is alarming in hospitals, has been compared to a slow-moving tsunami. The pathogens responsible for the most serious infections in humans include extended spectrum beta-lactamase (ESBL)-producing *Enterobacteriaceae*; carbapenem-resistant *Acinetobacter* species, methicillin-resistant *Staphylococcus aureus* (MRSA), vancomycin-resistant *Enterococcus faecalis* (VRE), and clarithromycin-resistant *Helicobacter pylori* [1,2].

In this context, Gram-positive strains of MRSA and VRE have been of particular clinical importance for years. Special attention is paid to hospital-acquired MRSA isolates, which pose a serious threat for patients due to their increasing prevalence and wide resistance to beta-lactam antibiotics and very often other, non-related groups of antibiotics. The MRSA phenotype constitutes almost 50% of all hospital *S. aureus* isolates [3,4] and is associated with the most severe, life-threatening pneumonia, bloodstream, and surgical site infections [5,6,7]. It is also not optimistic that a significant increase (up to 60%) in the number of community-acquired MRSA strains has been observed in recent years [8,9].

Vancomycin-resistant *Enterococci* (VRE) pose an equally big threat to the hospital environment as MRSA. In general, *Enterococci* are the leading factor of infections of wounds, the endocardium, as well as the urinary and circulatory system. They are also isolated in some cases of meningitis or pneumonia. The *Enterococci* constitute a significant challenge in the treatment of hospital patients due to their intrinsic resistance to several antibiotics, accumulation of mutations, and exogenous genes that confer additional resistance on these bacteria. Therefore, the infections caused by VRE strains (over 30% of patients) are difficult to treat and carry a high risk of death [10].

Although the eradication of Gram-positive bacteria poses a challenge for medicine, Gram-negative bacteria such as *Klebsiella* spp. and *Acinetobacter* spp., which are responsible for over 30% of hospital infections worldwide, currently constitute a bigger problem in the hospital setting [11]. Multidrug-resistant strains of *K. pneumoniae* are an important factor of the most severe hospital-acquired pneumonia, bacteremia, and urinary tract infections [12]. The alarming fact is that in the last decades, *Klebsiella* have developed resistance to most, or sometimes all, clinically available drugs. It reflects successful adaptation of the pathogen to the aggressive hospital environment predominated by invasive procedures. Clinical strains resistant to beta-lactam antibiotics, aminoglycosides, quinolones, tigecycline, and colistin pose a particular danger to hospitalised patients [13].

Among Gram-negative bacteria, *Acinetobacter* spp. demonstrates the highest potential for the spread among hospitalised patients due to its extraordinary ability of exogenous colonisation of the human body and high tolerance to harsh environmental conditions. *Acinetobacter baumannii*, which is associated with the most severe and difficult to treat infections such as bacteremia, respiratory, urinary tract infections, and surgical site infections, has been the most frequently recorded pathogen in Intensive Care Units worldwide [14,15,16]. The diseases caused by the multidrug-resistant strains of *A. baumannii* are associated with high mortality of 70% and 43% in ventilator-associated pneumonia (VAP) and bacteremia, respectively [17,18]. Due to its antimicrobial resistance and tendency to epidemic spread, *A. baumannii* can be considered one of the most dangerous hospital pathogens nowadays [19].

Although normally associated with nosocomial infections, *Helicobacter pylori* should be discussed in the context of therapeutic difficulties caused by other hospital-acquired pathogens. *H. pylori* is one of the most common human pathogens with an average incidence in the world exceeding 60% [20]. Colonisation with these bacteria in most cases leads to the development of chronic gastritis, while 10–15% and 1–3% of people, respectively, develop peptic ulcers and gastric cancers [21]. Therefore, the latest Maastricht V recommendations suggest the eradication of *H. pylori,* regardless of the presence of disease symptoms [22]. However, the problem is its high resistance to antibiotics, especially clarithromycin, which is the basic component of the first-line treatment [2]. *H. pylori* can survive for years in human stomach and accumulate mutations associated with resistance to various antibiotics, especially during monotherapies used against other pathogens [23,24]. For these reasons, it may become particularly dangerous to people subjected to long hospitalisation and treated for infections with MDR bacteria.

As the above facts show, the rapid development of antibiotic resistance and global spread of MDR pathogens make it necessary to look at potential alternative approaches to the attenuation of infections caused by these bacteria. Therefore, various systems of solutions that can alleviate the problem described in this article are proposed. One of such solutions is the search for combination therapies composed of a natural compound and an antibiotic, which would enhance antimicrobial therapy and enable lowering of the therapeutic doses. In our research, we focused on naringenin (5,7,4′-trihydroxyflavanone), which belongs to the class of flavonoid compounds known as flavanones. It can be commonly found in citrus fruits as aglycone, or in its glycoside form, naringin. Moreover, its *O*-alkyl derivatives are also found in rice leaves, e.g., sakuranetin (7-*O*-methylnaringenin) [25], or plants from the Boraginaceae family, e.g., 7,4′-di-*O*-methylnaringenin and 5-*O*-methylnaringenin [26]. Due to a wide range of its biological properties, such as antioxidative [27], anticancerogenic [28], antibacterial [29], or antifungal activity [30], naringenin and its derivatives are popular among the scientific community [31]. The chemical modification of naringenin and its *O*-alkyl derivatives, consisting of the incorporation of an oxime group, could enhance their antimicrobial properties.

Herein, we present the results of our research on the antibacterial activity of 18 *O*-alkyl derivatives of naringenin and their oximes. Four of them are being published for the first time. Due to their clinical relevance, antibiotic resistance status, and the fact that they cause serious human infections, we have chosen clinical strains of *S. aureus, E. faecalis, K. pneumoniae, A. baumannii*, and *H. pylori* as principle bacteria in the current work.

## 2. Results and Discussion

### 2.1. Chemistry

For the group of 18 compounds tested against priority pathogens, we have chosen 14 *O*-alkyl derivatives of naringenin and their oximes whose bacteriostatic activity against the non-MDR reference strains of bacteria has been proved in a previous work by Kozłowska et al. [32].

Accordingly, we selected seven *O*-alkyl derivatives of naringenin (**1a**–**7a**) and corresponding oximes (**1b**–**7b**) which had minimum inhibitory concentration (MIC) values within the range of 6.25–50.0 mg/mL against the strains of *S. aureus* and *B. subtilis*. Moreover, 7-*O*-methylnaringenin (sakuranetin) was chosen for the research due to the fact that it can be naturally found in rice. In addition, we decided to synthesise other, new *O*-alkyl derivatives of naringenin, containing six-carbon chains attached to the C-7 and/or C-4′ position (**8a** and **9a**), which have been further modified to their oximes (**8b** and **9b**). All derivatives used for antimicrobial assays are presented in Table 1.

**Scheme 1:**
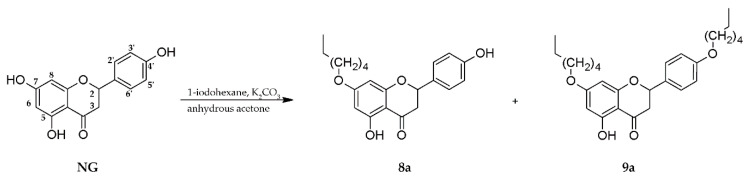
Synthesis of 7-*O*-hexylnaringenin (**8a**) and 7,4′-di-*O*-hexylnaringenin (**9a**).

Our previous studies did not show an unambiguous relationship between the length of the aliphatic chain and antimicrobial activity [32]. Therefore, the extension of our library by compounds that have not been described yet and the presentation of their biological activity is interesting and constitutes a novelty within the framework of this research. 7-*O*-hexylnaringenin (**8a**) and 7,4′-di-*O*-hexylnaringenin (**9a**) were obtained in one reaction of naringenin (NG) with 1-iodohexane in the presence of potassium carbonate, which is presented below (Scheme 1).

**Scheme 2:**
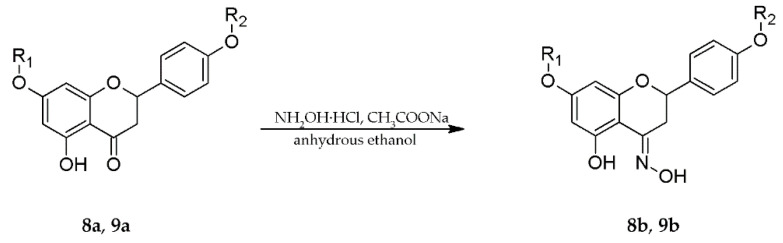
Synthesis of 7-*O*-hexylnaringenin oxime (**8b**) and 7,4′-di-*O*-hexylnaringenin oxime (**9b**); (**8a**, **8b**: R_1_ = CH_3_-(CH_2_)_5_, R_2_ = H; **9a, 9b**: R_1_ = R_2_ = CH_3_-(CH_2_)_5_).

Further modifications of compounds **8a** and **9a**, leading to obtaining their oximes, were performed in reaction with hydroxylamine hydrochloride and anhydrous sodium acetate with high yields of up to 99% (Scheme 2). The structure of the obtained novel compounds (**8a**, **9a, 8b**, **9b**) was confirmed by ^1^H- and ^13^C-NMR spectra, and the molecular weight was analysed with high-resolution mass spectrometry (HRMS).

The ^1^H-NMR spectrum confirmed the presence of one *O*-hexyl chain at C-7 position in 7-*O*-hexylnaringenin (**8a**) and its oxime (**8b**), and two *O*-hexyl chains at C-7 and C-4′ positions in 7,4′-di-*O*-hexylnaringenin and its oxime (**9a**, **9b**). Furthermore, in the case of **8b** and **9b**, a characteristic signal from the NOH group at 11.01 ppm was observed. Moreover, a downshifted signal on the ^13^C-NMR spectrum from 196.2 to 154.8 ppm and from 196.1 to 154.7 ppm confirmed the incorporation of an oxime moiety instead of the carbonyl group in both derivatives **8b** and **9b**, respectively.

### 2.2. Antimicrobial Studies

The microorganisms tested in this work represent a collection of priority clinical isolates of *Helicobacter pylori* (1), *Enterococcus faecalis* (2)*, Acinetobacter baumannii* (2)*, Staphylococcus aureus* (3)*,* and *Klebsiella pneumoniae* (3), which were acquired from gastrointestinal (1), urinary tract (2), and blood infections (3). All tested isolates exhibited resistance to antibiotics according to the EUCAST (The European Committee on Antimicrobial Susceptibility Testing) standards [33]. Their antimicrobial profiles, determined previously by the disc diffusion method or E-tests in routine clinical laboratory testing, are shown in Table 2.

The overall distribution of minimum inhibitory concentrations (MICs) and minimal bactericidal concentrations (MBCs) of the 18 compounds (Table 1) and antibiotics (vancomycin, colistin, gentamycin, imipenem, erythromycin, clarithromycin, levofloxacin, and metronidazole) used in standard eradication therapy are listed in Table 3. On basis of susceptibility testing and according to the EUCAST recommendations, we defined the strains of *E. faecalis* as VRE (vancomycin-resistant) and HLAR (high-level aminoglycoside resistant); *S. aureus* as MR or MRSA (methicillin-resistant *Staphylococcus aureus*) and *K. pneumoniae* as ESBL (extended spectrum beta-lactamase) producer.

The 18 naringenin derivatives and their oximes demonstrated potent but varied activity against all tested MDR isolates, with MICs/MBCs values ranging from 4 to > 1024 μg/mL/8 to > 1024 μg/mL for *H. pylori*, from 4 to > 1024 μg/mL/32 to > 1024 μg/mL for MRSA, and from 1 to > 1024 μg/mL/64 to > 1024 μg/mL for *E. faecalis* (Table 3). Reduced susceptibility to all tested compounds was observed in *K. pneumoniae* and *A. baumannii*, where the MIC and MBC values ranged from 512 to > 1024 μg/mL.

For *S. aureus*, the MICs of the 12 compounds showed relatively low values (4–64 μg/mL). However, MBC values for most of the compounds exceeded 1024 μg/mL, except for the **4b** (2 × MIC, 32 μg/mL), **7b** (16 × MIC, 128 μg/mL), and **8b** (32 × MIC, 128 μg/mL) compounds. In our opinion, the highest antibacterial potential against MRSA was shown by compound **4b**, the MIC and MBC parameters of which were equal to 16 μg/mL and 32 μg/mL, respectively (Table 3). In the case of *E. faecalis*, only six compounds showed high bacteriostatic activity, with MIC values ranging from 1 to 64 μg/mL. Most of them belong to the oximes of naringenin derivatives (group B, Table 3). Compared to *S. aureus*, *E. faecalis* showed lower susceptibility to the tested compounds, as also demonstrated by high MIC values (>1024 μg/mL). Only in the case of **4b** both MIC and MBC parameters were relatively low, with values amounting to 32 μg/mL and 64 μg/mL, respectively (Table 3).

The antimicrobial activity of flavonoids such as naringenin varies from compound to compound. Their inhibitory effect on microbial cells may be attributed to different mechanisms of action, including the damage of cytoplasmic membranes [34], inhibition of nucleic acid synthesis [35], and disruption of energy metabolism [36]. Interestingly, they are also indicated as factors affecting the process of biofilm formation [37], probably by interfering with the cellular communication system (quorum sensing).

It was demonstrated that individual species of bacteria exhibit different levels of susceptibility to naringenin, e.g., *Lactobacillus* spp. With an MIC value of 250 μg/mL [38], *S. aureus* with an MIC of 62.5 μg/mL, *Salmonella* Typhimurium and *E. coli* with MICs of 125 µg/mL [39]. The susceptibility of individual microorganisms to naringenin seems to be quite controversial, since there are also studies indicating the lack of its antimicrobial activity [29]. These discrepancies stem from a number of reasons. The most likely ones are the actual differences in susceptibility of specific strains and—which is worth emphasising—the research methodology itself, for example, various assay systems (initial inoculum size) or culture conditions. These factors may significantly affect the results of each experiment.

In the previous work by Kozłowska et al. [32], the structure and biological activity of seven novel *O*-alkyl derivatives of naringenin (**1a**–**7a**) and seven of their oximes (**1b**–**7b**) were demonstrated. They were tested against reference strains of *E. coli, S. aureus,* and *B. subtilis* from the American Type Culture Collection (ATCC). For example, 7,4′-di-*O*-isopropylnaringenin (**5a**) was inactive against *Escherichia coli*, *Bacillus subtilis,* and *S. aureus*, whereas 7,4′-di-*O*-isopropylnaringenin oxime (**5b**) exhibited activity against *B. subtilis* and *S. aureus* with MIC values equal to 6.25 μg/mL and 12.5 μg/mL, respectively [40]. The obtained results prompted us to carry out research on pathogens that are currently clinically relevant. Thus, we performed the current tests on strains of bacteria isolated from various human infections and resistant to antibiotics commonly recommended in standard therapy.

Their susceptibility/resistance to the 18 *O*-alkyl derivatives of naringenin and their oximes, which were tested in this research, can be presented in the following order, ranging from the most to the least sensitive strain: *H. pylori* (Gram-negative) > *S. aureus* (Gram-positive) > *E. faecalis* (Gram-positive) > *A. baumannii* (Gram-negative) > *K. pneumoniae* (Gram-negative). With regard to the structure—activity relationship, we cannot define an unequivocal strength of activity of *O*-alkyl derivatives and their oximes against all tested bacteria depending on the length of the alkyl chain. In the study of *H. pylori* (Gram-negative), we observed similar, strong activity of all mono-*O*-alkyl derivatives of naringenin (MIC values of 8 µg/mL, MBC values in a range of 8–32 µg/mL). Moreover, the MIC values for di-*O*-alkyl derivatives against this strain of bacteria were 2-fold higher, which indicates the better bacteriostatic activity of monosubstituted derivatives of naringenin (Table 3). The exception to this rule was compound **2b** (7,4′-di-*O*-methylnaringenin oxime), which similarly to compound **3b** (7-*O*-ethylnaringenin oxime) showed the highest activity against *H. pylori* with the MIC value of 4 µg/mL. The results observed for compounds **9a** and **9b** showed that the attachment of the six-carbon chain in the C-7 and C-4′ position of naringenin leads to a strong decrease in their activity against all tested Gram-negative bacteria. In the case of Gram-positive strains, the activity did not depend on the length of the chain, which made it impossible to determine the relationship. In general, we observed a higher antimicrobial activity of oxime derivatives (group B) than their naringenin counterparts (group A). Oximes **3b**, **4b**, **5b**, **7b**, and the newly synthesised oxime **8b**, which are described for the first time in this paper (Table 1), were the most active against *S. aureus* (MRSA) and *E. faecalis* (MIC 1–32 μg/mL). This high antibacterial activity is associated with the incorporation of an oxime group instead of carbonyl at the C-4 position in naringenin derivatives.

As in our previous work, the results of the current research confirmed the high resistance of Gram-negative rods to the tested compounds [32]. *K. pneumoniae* and *A. baumannii* showed reduced susceptibility against both groups of naringenin derivatives and their oximes. Here, most MIC values were equal to ≥1024 µg/mL. For these bacteria, the lowest MIC values, e.g., 512 µg/mL, were observed in the case of compounds **6b** and **8b**, and in the case of **1a**, **3b**, **4b**, **8b**, and **9b** against *K. pneumoniae* and *A. baumannii*, respectively. For both species, all tested compounds amounted to MBC (>1024 µg/mL) (Table 3). In contrast to Gram-positive microorganisms, the high antimicrobial resistance of Gram-negative bacteria, especially non-fermenting rods, stems mainly from the specific structure and low permeability of their cellular membranes [41].

Compared to the bacteria described above, the clarithromycin-resistant strain of *H. pylori* showed the highest susceptibility to most of the 18 compounds (Table 3)**.** Sixteen of them showed low MIC values relative to *H. pylori*, within the range of 4–16 µg/mL. Exceptions were compounds **9a** and **9b**, for which the MICs were >1024 µg/mL. The MBC values against *H. pylori* for the majority of the tested compounds were equal to MIC or 2 × MIC; however, in the case of **2a**, **3b**, and **7b**, they amounted to 4 × MIC. The lowest values of both above parameters against *H. pylori* were demonstrated for compound **2b** (MIC = 4 µg/mL and MBC = 8 µg/mL); therefore, the activity of this compound was determined in the following stages of the study. Low values of both MIC and MBC indicate that these compounds may be highly promising in the treatment of gastrointestinal infections caused by these bacteria.

To check the type of the interaction occurring between the tested antibiotics (gentamicin, erythromycin, clarithromycin, imipenem, vancomycin, levofloxacin, colistin, and metronidazole), naringenin derivatives, or their oximes, we used the checkerboard microdilution assay (Table 4). Our choice of compounds presented in Table 4 was made on the basis of their best activity (MIC, MBC) against the tested strains. Thus, we chose compounds **4b** and **8b** for further studies on staphylococci and enterococci and **2b** for studies on *H. pylori.* In the case of *A. baumannii* and *K. pneumoniae*, we chose compound **3b** (7-*O*-ethylnaringenin oxime). Although the activity (MIC and MBC values) of the other compounds (**1a**, **4b**, **8b**, **9b**) was similar to that of **3b**, our choice was based on the fact that oximes and short side chain compounds showed higher activity against *H. pylori*.

Fractional inhibitory concentrations (FICs) were calculated from the highest dilution of antibiotic combination permitting no growth, as determined by the optical density (OD) measurement. Synergy was defined by fractional inhibitory concentration index (FICI) ≤ 0.5 and antagonism by FICI > 4. FICI > 0.5 but ≤ 1 was considered as an additive effect, and FICI > 1 but ≤ 4 was considered as a neutral effect [42]. Table 4 presents the values of fractional inhibitory concentration (FIC) for antibiotics and naringenin compounds and the FICI calculated for each tested strain. Based on FICI, we observed the synergy against three bacterial isolates, e.g., *H. pylori*, *S. aureus,* and *E. faecalis*.

In the case of *H. pylori*, we noticed that **2b** showed synergistic or additive activity with all three tested antibiotics (clarithromycin, levofloxacin, metronidazole). An additive interaction was obtained for levofloxacin and metronidazole, enabling a 4-fold MIC reduction for both antibiotics (FICI = 0.75). In the case of clarithromycin, we observed a synergistic effect with **2b**, enabling a 4-fold MIC decrease in both components (FICI = 0.5), or an additive effect when the MICs of clarithromycin (CLR) and **2b** were lowered 8-fold and 2-fold, respectively (FICI = 0.625).

In the case of *E. faecalis*, the type of the interaction between the antibiotic and the compound depended on the antibiotic used. Positive interaction with vancomycin was observed for both **4b** (additive, FICI = 0.625) and **8b** (synergistic, FICI = 0.375), enabling a 2- and 8-fold reduction in vancomycin concentration, respectively. In the case of gentamicin, the interaction result was variable, and it was additive in the case of **4b** (FICI = 0.5625), causing a 16-fold decrease in the MIC of this antibiotic, while in the case **8b**, it was neutral (FICI = 2.0). Interaction with imipenem (FICI = 2.0) was not observed in either of the compounds.

The results of *S. aureus* testing were much more optimistic, since synergistic interactions have been detected for both compounds **8b** and **4b** used with an antibiotic. The use of **8b** reduced the MIC of both gentamicin and erythromycin by 4 times (FICI = 0.5). The use of **4b**, on the other hand, contributed to a 8-fold and 4-fold decrease in the MIC for gentamycin (FICI = 0.375) and erythromycin (FICI = 0.5), respectively.

No significant interactions were observed (FICI = 2.0) in the case of Gram-negative rods of *K. pneumoniae* and *A. baumannii*. The combination of **3b** with colistin (CPL) against *A. baumannii*, which resulted in an additive interaction, enabled us to obtain a 2-fold decrease in the MIC of the antibiotic and a 16-fold decrease in the MIC of **3b**, which constituted an exception.

The literature review provides numerous reports indicating that flavonoids, including naringenin, may potentiate the antimicrobial effect of various groups of antibiotics [36,43,44]. Despite the apparent non-selectivity of flavonoids in the enhancing of antibiotic activity, a closer analysis indicates representatives of beta-lactam antibiotics, glycopeptides, fluoroquinolones, nitroimidazoles, aminoglycosides, tetracyclines, and macrolides [36,42,43,44,45,46,47]. Knowledge of their target site on a bacterial cell [48] allowed us to conclude that flavonoids enhance the activity of antibiotics acting on the cell wall, genetic material, or protein synthesis. The presented data are consistent with the results obtained by our research group, as we were able to demonstrate a positive interaction between naringenin derivatives and macrolides (*H. pylori, S. aureus*), fluoroquinolones and nitroimidazoles (*H. pylori*), aminoglycosides (*E. faecalis, S. aureus*), glycopeptides (*E. faecalis*), and polypeptides (*A. baumannii*).

The target site of naringenin and its derivatives has not been clearly defined; however, the ability of this compound to disrupt fatty acid biosynthesis and increase membrane fluidity is indicated [49,50]. Alternatively, there are some data showing that naringenin is implicated in the inhibition of efflux pumps, which are proteins involved in the outflow of antibiotics from microbial cells [44,51]. It is also important to indicate that due to its strong antioxidant properties, naringenin has a high capacity to neutralise reactive oxygen species (ROS) [52], which are generated by some antibiotics, e.g., aminoglycosides, glycopeptides, or fluoroquinolones [53,54]. For example, it has been demonstrated with the use of gentamicin that various flavonoid compounds have the ability to reduce the oxidative stress-related toxicity of antibiotics against eukaryotic cells, without affecting their bactericidal activity [55]. Based on the obtained data, we can hypothesise about the ability of naringenin to induce increased the accumulation of antibiotics in microbial cells and influence the activity of various proteins included in different microbial molecular processes.

Another observation that is important to consider is the reduced sensitivity of Gram-negative rods (*K. pneumoniae, A. baumannii*) to the activity of naringenin derivatives and the lack of positive interactions with most of the tested antibiotics. A potential mechanism explaining the observed phenomenon is provided by Oh E. and Jeon B. [50] who demonstrated that lipopolysaccharide (LPS) may be responsible for decreasing the activity of different flavonoids, including naringenin. Although this study did not include naringenin derivatives, due to the non-specificity of this process, it cannot be ruled out that similar phenomenon occurs also in the case of naringenin derivatives studied in the present work. Since LPS is a structural component typical of Gram-negative bacteria [56], it seems that it may play an important role in lowering the sensitivity of this group of bacteria to the naringenin derivatives we tested. *H. pylori* is also a representative of Gram-negative rods with strongly modified LPS, which determines the resistance of this bacterium to the immune system [57], but it may, in our opinion, increase its sensitivity to flavonoids. In the future, this hypothesis will be verified by our research group.

**Figure 1:**
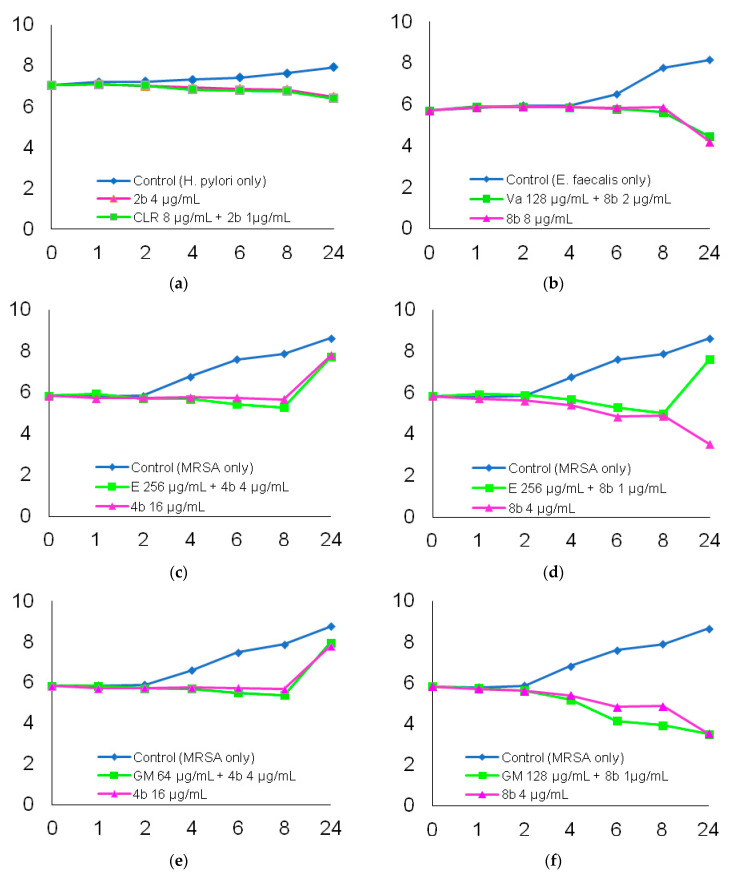
Time-kill kinetics of the oximes of naringenin derivatives against clinical strains of: (**a**) *H. pylori*, (**b**) *E. faecalis*, (**c**–**f**) *S. aureus* (MRSA). Bacterial counts were determined at time zero and after 1, 2, 4, 6, 8, and 24 h from incubation. Bacterial cultures were only used as controls. The values are the averages of three independent determinations. *Y*-axis: cell viability [log_10_CFU/mL]; *X*-axis: time [hours]; MRSA (methicillin-resistant *S. aureus*); CLR (clarithromycin); VA (vancomycin); GM (gentamycin); E (erythromycin); **2b** (7,4′-di-*O*-methylnaringenin oxime); **4b** (7-*O*-isopropylnaringenin oxime); **8b** (7-*O*-hexylnaringenin oxime).

We also performed kill curves for *H. pylori, E. faecalis,* and *S. aureus* to determine the time-dependent bacteriostatic/bactericidal properties of the combinations of selected antibiotics and the most active compounds in synergistic concentrations (Figure 1).

The analysis of time-dependent activity of **2b** on *H. pylori* showed a bacteriostatic effect of this oxime, both alone and in combination with clarithromycin (Figure 1a). We noticed that after a 24-h-long incubation, the concentrations corresponding to the MIC of **2b** and FICI = 0.5 with clarithromycin (synergistic interaction) decreased the viability of *H. pylori* by about 1.5 log_10_ relative to the control.

Similarly, a bacteriostatic effect (<2logs of killing) was observed in tests of the activity of **8b** against *E. faecalis*, alone and in combination with vancomycin. After 24 h of growth in the presence of vancomycin and **8b**, the assays showed 1.8 logs of killing for *E. faecalis*, relative to the control, which corresponded to a reduction in CFU (colony-forming unit)/mL in the 4-log_10_ range. This is corroborated by the MIC of this substance and FICI = 0.375 (synergistic interaction) (Figure 1b). The combination of compound **4b** with erythromycin (Figure 1c) and gentamicin (Figure 1e) showed bacteriostatic effect within the range of 7.7- to 7.9-log_10_ reduction in CFU/mL of *S. aureus*. This corresponded to the 1.1 logs of killing compared to the control, for both erythromycin and gentamicin. This observation was confirmed by the MIC of **4b**, but it was not consistent with the results of the checkerboard assay, where we obtained synergistic activity with the tested antibiotics. Among the synergistic concentrations used, bacteriostatic activity against *S. aureus* was also observed in the case of **8b** oxime, both on its own and in combination with erythromycin (Figure 1d) and gentamicin (Figure 1f). In combination with antibiotics, the substance led to a reduction in CFU/mL in the 7.6-(1.1 logs of killing) and 3.5-(2.5 logs of killing) log_10_ range for erythromycin and gentamicin, respectively. This was compatible with the MIC, but not with the results of the checkerboard assay, where FICI = 0.5 (for erythromycin) and 0.375 (for gentamicin). Flavonoids are reported to be substances exhibiting mainly bacteriostatic activity against Gram-positive and Gram-negative bacteria [58]. They are known not to kill microbial cells, but they do induce the formation of pseudomulticellular aggregates, which results in a decrease in the number of detected cells (CFU/mL) [59]. Nevertheless, in our study, MBC values for the tested compounds were many times higher than the MICs and exceeded 1024 µg/mL. This indicates that it would not be possible to achieve a bactericidal effect in vivo conditions. Exceptions were compounds **4b** and **8b**, which significantly reduced the survival of *S. aureus* and *E. faecalis.* The studies conducted by Cushnie et al. [59] showed that the aggregation of bacterial cells can be an effect of cytoplasmic membrane damage, but it may also be caused by hydrophobic regions interacting with one another. To some extent, it may explain the bactericidal effect of the most hydrophobic 7-*O*-hexylnaringenin oxime (**8b**). However, it is difficult to explain the equally strong activity of compound **4b** (7-*O*-isopropylnaringenin oxime), which is less hydrophobic than compound **8b**. The MBC value of compound **4b** was twice as low against *S. aureus* than *E. faecalis.* In the case of compound **8b**, we observed a similar situation, but the effect against *S. aureus* was 8 times stronger compared to the effect on *E. faecalis*.

In this study, we also observed an increase in *S. aureus* inoculum in the combination of **4b** + E, **4b** + GM, and **8b** + E at a 24 h incubation time point. Domaracki et al. [60] indicate that not all compounds for which synergy has been demonstrated in the checkerboard assay also show synergistic activity (≥2 logs of killing) in the time-killing studies. According to the data from the literature [61], our result may indicate that the *S. aureus* strain studied by us has the ability to remove antibiotics from the growth medium by binding them through cell well structures. Therefore, re-administration halfway through the incubation of the time-kill assay is suggested.

## 3. Materials and Methods

### 3.1. Strains and Culture Conditions

Five opportunistic pathogens of *Enterococcus faecalis* 37VRE, *Staphylococcus aureus* KJ, *Acinetobacter baumannii* 2800, *Klebsiella pneumoniae* N111, and *Helicobacter pylori* 7189 type were used in the study. All strains were hospital-acquired and obtained from the collection of the Department of Microbiology at the Wroclaw Medical University.

The bacterial cultures were kept at −80 °C. The overnight cultures (except *H. pylori*) were prepared in the Tryptic Soy Broth (TSB, OXOID, Basingstoke, UK) and incubated in a shaking incubator (MaxQ ^TM^ 6000 incubator shaker, Thermo Scientific, Waltham, MA, USA) at 125 rpm and 37 °C. For every experiment, we prepared 18–20 h subcultures on the Tryptic Soy Agar (TSA, OXOID, Basingstoke, UK), which were then transferred into 3 mL of fresh Mueller Hinton broth (MHB, OXOID, Basingstoke, UK). The density of each culture was adjusted to 10^8^ CFU/mL and then diluted to 10^6^ CFU/mL (primary inoculum). In the case of *H. pylori*, after thawing, the bacteria were plated on Columbia agars (Difco, Lublin, Poland) with 7% hemolysed horse blood, and they were incubated for 3–5 days under microaerophilic conditions (Genbox micro aer kits, BioMerieux, Marcy I’Etoile, France) at 37 °C. Then, suspensions of *H. pylori* with a density of 10^8^ CFU/mL were prepared in the brain-heart infusion broth (BHI, OXOID, Basingstoke, UK) with 7% fetal calf serum (FCS, Gibco, Paisley, UK) and forwarded to the next stage of the research.

### 3.2. Antimicrobial Agents

Vancomycin (VA), colistin (COL), gentamycin (GM), imipenem (IMP), aztreonam (ATM), erythromycin (E), clarithromycin (CLR), levofloxacin (LEV), and metronidazole (MTZ) were obtained from Sigma-Aldrich (St. Louis, MO, USA). The test solutions were prepared before usage.

### 3.3. Chemistry

#### 3.3.1. Chemicals

Naringenin and 1-iodohexane were purchased from Sigma-Aldrich Co. (St. Louis, MO, USA); hydroxylamine hydrochloride was purchased from LOBA Feinchemie GmbH (Fischamed, Austria); anhydrous sodium acetate and potassium carbonate were purchased from Chempur (Piekary Śląskie, Poland). Anhydrous solvents for reactions (acetone and ethanol) were prepared according to the standard procedures. All organic solvents were of analytical grade.

#### 3.3.2. Analysis

The thin layer chromatography (TLC) analysis using silica gel-coated aluminum sheets with a fluorescent indicator (DC-Alufolien, Kieselgel 60 F_254_; Merck, Darmstadt, Germany) was performed in order to monitor the progress of the reactions. The TLC plates were sprayed with a solution of 1% Ce(SO_4_)_2_ and 2% H_3_[P(Mo_3_O_10_)_4_] in 5% H_2_SO_4_ and heated to visualise the product of the reaction. The separation and purification of products was performed by liquid column chromatography using silica gel (Kieselgel 60, 230–400 mesh, Merck, Darmstadt, Germany). The structures of the obtained products were determined using ^1^H and ^13^C nuclear magnetic resonance (NMR) spectra, which were recorded on a Bruker Avance^TM^600 MHz spectrometer (Bruker, Billerica, MA, USA). Samples for the NMR analysis were prepared using deuterated solvents: acetone-*d_6_* and chloroform-*d* (Supplementary Materials, Figures S1–S8). The spectroscopic data of compounds **1a**–**7a** and **1b**–**7b** were described in our previous work [30,32].

To confirm the molar masses of the synthesised derivatives, high-resolution electrospray ionization mass spectrometry (ESI-MS) spectra were measured on a Bruker ESI-Q-TOF Maxis Impact Mass Spectrometer (Bruker, Billerica, MA, USA). Regarding the direct infusion of ESI-MS parameters, mass spectrometer was operated in positive ion mode with the potential between the spray needle and the orifice equal to 4.0 kV, nebulizer pressure equal to 0.4 bar, and drying gas flow rate equal to 3.0 L/min at 180 °C. The sample flow rate was 3.0 µL/min. Ionisation mass spectra were collected within the range of *m*/*z* 50–1350.

The melting points (uncorrected) were determined with the use of a Boetius apparatus (Carl Zeiss, Jena, Germany).

#### 3.3.3. Synthesis of 7-O-hexylnaringenin (**8a**) and 7,4′-di-O-hexylnaringenin (**9a**)

Naringenin (1.84 mmol) was dissolved in the smallest amount of anhydrous acetone (8 mL). Then, anhydrous potassium carbonate (2.75 mmol) and 1-iodohexane (9.18 mmol) were added. The mixture was stirred for 45 h at 45 °C on a magnetic stirrer. After that, the acetone was evaporated, and water (30 mL) as well as a saturated solution of sodium chloride (40 mL) were added. The extraction was carried out using diethyl ether (3 × 50 mL). The collected and combined organic fractions were dried over sodium sulfate and concentrated on a vacuum evaporator. As a result of the reaction, two *O*-alkyl derivatives of naringenin (**8a** and **9a**) were obtained, which were separated by glass column chromatography (ϕ = 4 cm) using a mixture of hexane/methylene chloride/ethyl acetate 5:1:1 (*v*:*v*:*v*) as the eluent. The spectroscopic and physical data of compounds **8a** and **9a** are described below.

7-*O*-Hexylnaringenin (**8a**), white powder, yield 62.6% (0.410 g), m.p. 120–121 °C; ^1^H-NMR (600 MHz, Chloroform-*d*) δ [ppm]: 12.01 (s, 1H, OH-5), 7.37–7.29 (m, 2H, AA’BB’, H-2′, H-6′), 6.92–6.84 (m, 2H, AA’BB’, H-3′, H-5′), 6.06 (d, *J* = 2.3 Hz, 1H, H-6), 6.03 (d, *J* = 2.3 Hz, 1H, H-8), 5.42 (br s, 1H, OH-4′), 5.34 (dd, *J* = 13.0, 3.0 Hz, 1H, H-2), 3.96 (t, *J* = 6.6 Hz, 2H, -CH_2_-), 3.08 (dd, *J* = 17.1, 13.0 Hz, 1H, H-3_a_), 2.78 (dd, *J* = 17.1, 3.0 Hz, 1H, H-3_b_), 1.78–1.73 (m, 2H, -CH_2_-), 1.45–1.39 (m, 2H, -CH_2_-), 1.35–1.30 (m, 4H, 2x-CH_2_-), 0.91 (t, *J* = 7.0 Hz, 3H, -CH_3_); ^13^C-NMR (150 MHz, Chloroform-*d*) δ [ppm]: 196.2 (C=O), 167.8, 164.2, 163.0, 156.3, 130.7, 128.1, 115.8, 103.1, 95.7, 94.8, 79.1, 68.7, 43.3, 31.6, 29.0, 25.7, 22.7, 14.1; HRMS (*m*/*z*): [M + H]^+^ calcd. for C_21_H_25_O_5_, 357.1697; found 357.1694.

7,4′-Di-*O*-hexylnaringenin (**9a**), light yellow powder, yield 25.5% (0.206 g); m.p. 61–62 °C; ^1^H-NMR (600 MHz, Chloroform-*d*) δ [ppm]: 12.02 (s, 1H, OH-5), 7.39–7.32 (m, 2H, AA’BB’, H-2′, H-6′), 6.97–6.91 (m, 2H, AA’BB’, H-3′, H-5′), 6.05 (d, *J* = 2.3 Hz, 1H, H-6), 6.03 (d, *J* = 2.3 Hz, 1H, H-8), 5.35 (dd, *J* = 13.0, 3.0 Hz, 1H, H-2), 3.97 (t, *J* = 6.6 Hz, 2H, -CH_2_-), 3.95 (t, *J* = 6.6 Hz, 2H, -CH_2_-), 3.09 (dd, *J* = 17.1, 13.0 Hz, 1H, H-3_a_), 2.78 (dd, *J* = 17.1, 3.0 Hz, 1H, H-3_b_), 1.81–1.73 (m, 4H, 2x-CH_2_-), 1.49–1.40 (m, 4H, 2x-CH_2_-), 1.37–1.30 (m, 8H, 4x-CH_2_-), 0.94–0.87 (m, 6H, 2x-CH_3_); ^13^C-NMR (150 MHz, Chloroform-*d*) δ [ppm]: 196.1 (C=O), 167.7, 164.2, 163.0, 159.7, 130.3, 127.8, 114.9, 103.1, 95.7, 94.7, 79.1, 68.7, 68.3, 43.3, 31.7, 31.6, 29.3, 29.0, 25.8, 25.7, 22.7, 22.7, 14.2, 14.1; HRMS (*m*/*z*): [M + H]^+^ calcd. for C_27_H_37_O_5_, 441.2636; found 441.2630.

#### 3.3.4. Synthesis of Oximes (**8b**, **9b**)

The *O*-alkyl derivative of naringenin was dissolved in anhydrous ethanol (5 mL), and hydroxylamine hydrochloride and anhydrous sodium acetate were subsequently added in a molar ratio of 1:3:3, respectively. The reaction was carried out with a magnetic stirrer at 40–50 °C for 5 h (compound **8b**) or 25 h (compound **9b**). After that time, the mixture was poured into ice water, and the precipitated crystals were collected. The crude products were purified by glass column chromatography (ϕ = 2 cm) using a mixture of chloroform:methanol 96:4 (*v*:*v*) (compound **8b**) or 100:0.2 (*v*:*v*) (compound **9b**) as the eluent. The spectroscopic and physical data of compounds **8b** and **9b** are described below.

7-*O*-Hexylnaringenin oxime (**8b**), white powder, yield 79.1% (0.206 g), m.p. 187–189 °C; ^1^H-NMR (600 MHz, Acetone-*d*_6_) δ [ppm]: 11.01 (s, 1H, NOH), 7.43–7.33 (m, 2H, AA’BB’, H-2′, H-6′), 6.94–6.83 (m, 2H, AA’BB’, H-3′, H-5′), 6.05 (d, *J* = 2.3 Hz, 1H, H-6), 6.03 (d, *J* = 2.3 Hz, 1H, H-8), 5.07 (dd, *J* = 12.0, 3.1 Hz, 1H, H-2), 3.96 (t, *J* = 6.5 Hz, 2H, -CH_2_-), 3.46 (dd, *J* = 17.2, 3.1 Hz, 1H, H-3_a_), 2.79 (dd, *J* = 17.2, 12.0 Hz, 1H, H-3_b_), 1.78–1.70 (m, 2H, -CH_2_-), 1.48–1.42 (m, 2H, -CH_2_-), 1.38–1.30 (m, 4H, 2x-CH_2_-), 0.90 (t, *J* = 7.0 Hz, 3H, -CH_3_); ^13^C-NMR (150 MHz, Acetone-*d*_6_) δ [ppm]: 162.9, 160.6, 159.4, 158.4, 154.8 (C=NOH), 131.7, 128.8, 116.1, 99.1, 96.6, 95.1, 77.4, 68.7, 32., 30.3, 26.4, 23.3, 14.3; HRMS (*m*/*z*): [M + H]^+^ calcd. for C_21_H_26_NO_5_, 372.1805; found 372.1807.

7,4′-Di-*O*-hexylnaringenin oxime (**9b**), white powder, yield 98.6% (0.255 g), m.p. 75–77 °C; ^1^H-NMR (600 MHz, Acetone-*d*_6_) δ [ppm]: 11.01 (s, 1H, NOH), 10.40 (s, 1H, OH-5), 7.48–7.43 (m, 2H, AA’BB’, H-2′, H-6′), 7.00–6.96 (m, 2H, H-3′, H-5′), 6.05 (d, *J* = 2.3 Hz, 1H, H-6), 6.04 (d, *J* = 2.3 Hz, 1H, H-8), 5.11 (dd, *J* = 11.9, 3.2 Hz, 1H, H-2), 4.02 (t, *J* = 6.5 Hz, 2H, -CH_2_-), 3.97 (t, *J* = 6.5 Hz, 2H, -CH_2_-), 3.47 (dd, *J* = 17.2, 3.2 Hz, 1H, H-3_a_), 2.81 (dd, *J* = 17.2, 11.9 Hz, 1H, H-3_b_), 1.80–1.72 (m, 4H, 2x-CH_2_-), 1.50–1.44 (m, 4H, 2x-CH_2_-), 1.37–1.31 (m, 8H, 4x-CH_2_-), 0.92–0.89 (m, 6H, 2x-CH_3_); ^13^C-NMR (150 MHz, Acetone-*d*_6_) δ [ppm]: 163.0, 160.6, 160.2, 159.3, 154.7 (C=NOH), 132.7, 130.3, 128.7, 115.3, 99.2, 96.6, 95.1, 77.2, 68.7, 68.6, 32.3, 32.3, 30.3, 26.5, 26.4, 23.3, 23.3, 14.3, 14.3; HRMS (*m*/*z*): [M + H]^+^ calcd. for C_27_H_38_NO_5_, 456.2744; found 456.2745.

### 3.4. Susceptibility Testing

The MIC of each antibiotic was determined with the use of the broth microdilution method in accordance with the standards of the European Antimicrobial Susceptibility Testing Committee (EUCAST) [33].

A number of dilutions of twice concentrated compounds in 100 µL MHB in concentrations ranging from 2 to 2048 µg/mL and twice concentrated DMSO (Chempur, Piekary Śląskie, Poland) (0.02–20.48%) were prepared in 96-well titration plates in three repetitions. The stock solution (20 mg/mL) for the *O*-alkyl derivatives of naringenin and their oximes **1a**–**9a**, **1b**–**9b** was dissolved in DMSO, for vancomycin, imipenem, and colistin (Sigma-Aldrich, St. Louis, MO, USA) in water (Polpharma SA, Starogard Gdański, Poland), and for aztreonam, erythromycin, clarithromycin, levofloxacin, and metronidazole (Sigma-Aldrich, St. Louis, MO, USA) in DMSO. One-hundred microliters of bacterial culture in MHB (10^6^ CFU/mL) were added to 100 µL MHB with an adequate concentration of the tested compounds, obtaining the final concentrations of the tested preparations in the well from 1 to 1024 µg/mL and an initial inoculum of 5 × 10^5^ CFU/mL. A number of compound dilutions were prepared in the same way as compound background, adding 100 µL of clear MHB to twice-concentrated compounds in MHB. For *H. pylori*, the series of dilutions were prepared in 12-well plates containing 1 mL of BHI + FCS and 10^7^ CFU/mL of bacteria, which were obtained by adding 0.5 mL of BHI + FCS with twice the concentration of the tested compound, 0.4 mL of BHI + FCS and 0.1 mL of BHI + FCS containing 10^8^ CFU/mL of bacteria. As mentioned above, the study on *H. pylori* involved concentrations of substances within the range of 1–1024 µg/mL and testing of the turbidity of the substance (without the presence of bacteria) as a control.

Growth controls were as follows: a bacterial culture in MHB and a bacterial culture in MHB with adequate DMSO concentration (0.01–10.24%), or BHI with 7% serum and a bacterial culture with the above concentrations of DMSO for *H. pylori*.

Titration plates were incubated for 18 h for the *S. aureus*, *A. baumannii,* and *K. pneumoniae* strains, 24 h and 48 h for the *E. faecalis* strain, and 72 h for the *H. pylori* strain. Subsequently, the optical density (OD_600nm_) was read with the use of a titration plate reader (ASYS UVM340, BIOCHROM Ltd., Cambridge, UK). The average OD_600_ value of compounds alone was subtracted from the average OD600 value of bacteria with the tested compounds. The obtained result was compared with the OD600 average of clean MHB substrate. The first OD_600_ value not exceeding the OD_600_ of clean substrate was adopted as the MIC value.

To determine the minimal bactericidal concentration (MBC), 10 μL of bacterial suspension from each well of the microtiter plate was inoculated onto a TSA plate and incubated for 24 h at 37 °C. The MBC value was considered to be the lowest concentration at which no bacterial growth was detected.

### 3.5. Checkerboard Assay

All five drug-resistant strains of *S. aureus, A. baumannii, K. pneumoniae, E. faecalis,* and *H. pylori* were used in the study due to their resistance to the above antibiotics determined by the MIC microdilution assay. The MICs of the antibiotics and the selected naringenin derivatives in combination were determined using the two-dimensional checkerboard microdilution assay.

Only compounds **2b**, **3b**, **4b**, **6b,** and **8b** showing the strongest activity inhibiting the growth of the studied microbes were selected for the study. A 96-well plate or a panel of six 12-well plates (for *H. pylori*) contained increasing concentrations of the antibiotic on the *x*-axis and increasing concentrations of the tested compound on the *y*-axis (the ranges of the tested concentrations are provided in Table 3). Studies were conducted on the Mueller Hinton broth substrate and a final inoculum of 4–6 × 10^5^ CFU/mL. The cultures were incubated for 18 h at 37 °C. In the case of *H. pylori*, tests were carried out in BHI + FCS with a bacterial suspension of 10^7^ CFU/mL. Such bacteria were incubated for three days in microaerophilic conditions at 37 °C and 100 rpm. For all studied strains, after the period of incubation, an increase or lack thereof was visually read, and the OD_600_ optical density was measured using the ASYS UVM340 microplate reader. After the plotting of both readings, the MIC in the combination was determined.

Partial FIC values of the antibiotic and naringenin derivative, which enabled the assessment of the interaction between the tested compounds by determining the FIC_INDEX_, were determined using the method.

The fractional inhibitory concentration index (FIC_INDEX_) was calculated as a sum of the MIC of each compound when used in combination, divided by the MIC of the compound used alone.

A/MIC_A_ + B/MIC_B_ = FIC_A_ + FIC_B_ = FIC_INDEX_ (1)

A (antibiotic) and B (compound) constitute the MIC of each component in the combination, and MIC_A_ and MIC_B_ determine the MIC of single component).

### 3.6. Time-Kill Analysis

The study was performed for three isolates of *S. aureus, E. faecalis,* and *H. pylori* at concentrations based on the MIC determined during the checkerboard testing. Bacteria were grown overnight at 37 °C in MHB and then diluted to 0.5 using McFarland standard turbidity. A bacterial culture in 20 mL MHB constituted the control group.

The following combinations were tested in the study:

(a) for *S. aureus*: the gentamicin (64 µg/mL) + compound **4b** (4 µg/mL) combination, as well as the gentamicin (128 µg/mL) + compound **8b** (1 µg/mL) combination and the erythromycin (256 µg/mL) + compound **4b** (4 µg/mL), the erythromycin (256 µg/mL) + compound **8b** (1 µg/mL) combination;
(b) for *E. faecalis*: the vancomycin (128 µg/mL) + compound **8b** (2 µg/mL) combination;
(c) for *H. pylori*: the clarithromycin (8 µg/mL) + compound **2b** (1 µg/mL) combination.

For *S. aureus* and *E. faecalis,* 10 mL of bacterial culture was added to 10 mL of twice concentrated antibiotic solution with the tested compound, obtaining an initial concentration of suspension equal to 4–6 × 10^5^ CFU/mL in MHB. For *H. pylori*, 0.4 mL of BHI + FCS and 0.1 mL of BHI + FCS containing 10^8^ CFU/mL of bacteria were added to 0.5 mL of BHI + FCS with twice the concentration of the tested compound, obtaining the desired concentrations of compounds and bacterial density. After 1 h, 2 h, 4 h, 6 h, 8 h and 24 h of incubation at 37 °C, a number of solutions were performed for the study and control groups, and 50 µL in three repetitions were plated out from 10^0^–10^−6^ dilutions on TSA or Columbia agar substrate with 7% horse blood. After 24 h of incubation (for *S. aureus* and *E. faecalis*) or 72 h (for *H. pylori*) at 37 °C, the grown bacterial colonies were counted, and CFU/mL was calculated. The results are presented in the form of charts, depicting the changes occurring in CFU/mL in time.

## 4. Conclusions

In summary, we evaluated the antimicrobial activity of 18 *O*-alkyl derivatives of naringenin and their oximes. To our best knowledge, compounds **8a**, **9a**, **8b**, and **9b** are novel and have never before been described in scientific reports. Oxime derivatives showed higher antimicrobial activity against clinically relevant strains of *Enterococcus faecalis*, *Staphylococcus aureus*, *Acinetobacter baumannii*, *Klebsiella pneumoniae,* and *Helicobacter pylori* than their naringenin counterparts. The increased activity stems from the incorporation of an oxime group instead of carbonyl at the C-4 position in naringenin derivatives. Gram-positive cocci of *S. aureus* and *E. faecalis* were more susceptible to the tested compounds than Gram-negative rods of *A. baumannii* and *K. pneumoniae*. However, it was interesting to discover that another Gram-negative, spiral bacteria of *H. pylori* type exhibited the highest susceptibility to most of our compounds, as evidenced by the lowest MIC and MBC values. These observations allow us to set a new research goal, which will explain the mechanism of interaction between these promising compounds and the cells of pathogenic microorganisms.

## Figures and Tables

**Table 1:** *O*-alkyl derivatives of naringenin (**1a**–**9a**) and their oximes (**1b**–**9b**).

No.	*O*-alkyl Derivatives	No.	Oximes
**1a**	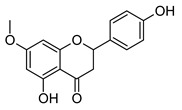	**1b**	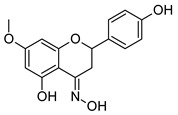
	7-*O*-methylnaringenin		7-*O*-methylnaringenin oxime
**2a**	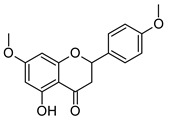	**2b**	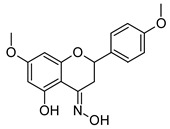
	7,4′-di-*O*-methylnaringenin		7,4′-di-*O*-methylnaringenin oxime
**3a**	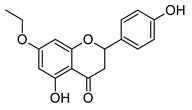	**3b**	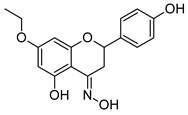
	7-*O*-ethylnaringenin		7-*O*-ethylnaringenin oxime
**4a**	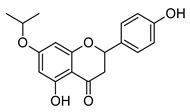	**4b**	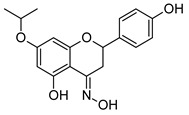
	7-*O*-isopropylnaringenin		7-*O*-isopropylnaringenin oxime
**5a**	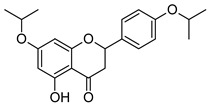	**5b**	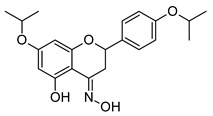
	7,4′-di-*O*-isopropylnaringenin		7,4′-di-*O*-isopropylnaringenin oxime
**6a**	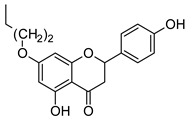	**6b**	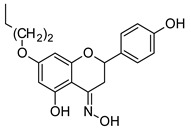
	7-*O*-butylnaringenin		7-*O*-butylnaringenin oxime
**7a**	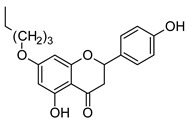	**7b**	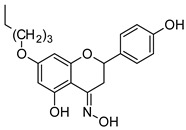
	7-*O*-pentylnaringenin		7-*O*-pentylnaringenin oxime
**8a**	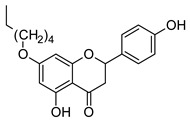	**8b**	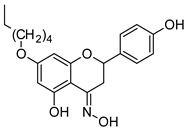
	7-*O*-hexylnaringenin		7-*O*-hexylnaringenin oxime
**9a**	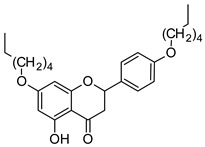	**9b**	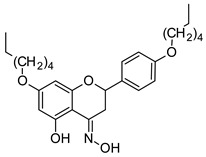
	7,4′-di-*O*-hexylnaringenin		7,4′-di-*O*-hexylnaringenin oxime

**Table 2:** Antimicrobial profiles determined by disc diffusion method.

Strain	Antimicrobial Resistance Profile
*Helicobacter pylori* 7189	CLR ^R^, MTZ ^S^, LEV ^S^
*Staphylococcus aureus* KJ	FOX ^R^, E ^R^, CLD ^R^, GM ^S^, CIP ^R^,TET ^S^, SXT ^S^, LZD ^S^
*Enterococcus faecalis* 37VRE	VA ^R^, GM ^R^, AMP ^S^, IMP ^S^, TEC ^S^
*Klebsiella pneumoniae* N111	GM ^R^, TOB ^R^, CIP ^R^, CTX ^R^, CAZ ^R^, CXM ^R^, AMC ^R^, ATM ^R^, FEP ^R^, SAM ^R^, TZP ^R^, IMP ^S^, MEM ^S^, DOR ^S^, CPL ^S^, AKN ^S^
*Acinetobacter baumannii* 2800	IMP ^R^, MEM ^R^, CIP ^R^, GM ^R^, TOB ^R^, LEV ^R^, FEP ^R^, CAZ ^R^, PIP ^R^, TZP ^R^, AKN ^R^, SXT ^R^, CPL ^S^

^R^ resistant; ^S^ susceptible; AMC (amoxicillin/clavulanic acid), AMP (ampicillin), AKN (amikacin), ATM (aztreonam), CAZ (ceftazidime), CLR (clarithromycin), CIP (ciprofloxacin), CLD (clindamycin), CPL (colistin), CTX (cefotaxime), CXM (cefuroxime), DOR (doripenem), E (erythromycin), FEP (cefepime), FOX (cefoxitin), GM (gentamicin), IMP (imipenem), LEV (levofloxacin), LZD (linezolid), MEM (meropenem), MTZ (metronidazole), PIP (piperacillin), SAM (ampicillin/sulbactam), TEC (teicoplanin), TET (tetracycline), TOB (tobramycin), TZP (piperacillin/tazobactam), SXT (co-trimoxazole), VA (vancomycin).

**Table 3:** Minimum inhibitory concentrations (MICs) and minimal bactericidal concentrations (MBCs) of naringenin derivatives (**1a**–**9a**) and their oximes (**1b**–**9b**) and selected antibiotics, tested against clinical isolates.

**MIC (MBC) [µg/mL]**					
	***H. pylori***	***S. aureus***	***E. faecalis***	***K. pneumoniae***	***A. baumannii***
**Compounds**					
**1a**	8 (8)	32 (>1024)	512 (>1024)	>1024 (>1024)	512 (>1024)
**2a**	16 (64)	>1024 (>1024)	512 (>1024)	>1024 (>1024)	>1024 (>1024)
**3a**	8 (16)	64 (>1024)	512 (>1024)	>1024 (>1024)	1024 (>1024)
**4a**	8 (8)	32 (>1024)	64 (>1024)	>1024 (>1024)	>1024 (>1024)
**5a**	16 (16)	512 (>1024)	512 (>1024)	>1024 (>1024)	1024 (>1024)
**6a**	8 (8)	8 (>1024)	>1024 (>1024)	>1024 (>1024)	1024 (>1024)
**7a**	8 (16)	32 (>1024)	512 (>1024)	>1024 (>1024)	1024 (>1024)
**8a**	16 (32)	1024 (>1024)	128 (>1024)	>1024 (>1024)	>1024 (>1024)
**9a**	>1024 (>1024)	512 (>1024)	>1024 (>1024)	>1024 (>1024)	>1024 (>1024)
**1b**	8 (16)	32 (>1024)	1024 (>1024)	>1024 (>1024)	1024 (>1024)
**2b**	4 (8)	512 (>1024)	1024 (>1024)	>1024 (>1024)	1024 (>1024)
**3b**	4 (32)	16 (>1024)	1 (>1024)	>1024 (>1024)	512 (>1024)
**4b**	8 (16)	16 (32)	32 (64)	>1024 (>1024)	512 (>1024)
**5b**	16 (16)	8 (>1024)	2 (>1024)	>1024 (>1024)	1024 (>1024)
**6b**	8 (16)	4 (>1024)	512 (>1024)	512 (>1024)	1024 (>1024)
**7b**	8 (32)	8 (128)	8 (>1024)	>1024 (>1024)	1024 (>1024)
**8b**	16 (16)	4 (128)	8 (>1024)	512 (>1024)	512 (>1024)
**9b**	>1024 (>1024)	512 (>1024)	512 (>1024)	>1024 (>1024)	512 (>1024)
**Antibiotics**					
GM	-	512 (>512)	>1024 (>1024)	8 (256)	>512 (>512)
VA	-	-	512 (>1024)	-	-
E	-	1024 (>1024)	4 (16)	-	-
IMP	-	-	-	0.5 (1)	32 (32)
CPL	-	-	-	1 (2)	2 (2)
CLR	32 (64)	-	-	-	-
LEV	0.5 (0.5)	-	-	-	-
MTZ	8 (16)	-	-	-	-

GM (gentamicin), VA (vancomycin), E (erythromycin), IMP (imipenem), CPL (colistin), CLR (clarithromycin), LEV (levofloxacin), MTZ (metronidazole).

**Table 4:** The interaction of the selected naringenin derivatives and their oximes with antibiotics used in *H. pylori, S. aureus, E. faecalis, K. pneumoniae,* and *A. baumannii* eradication therapies.

Strain	Combination	FIC_ANTIBIOTIC_	FIC_COMPOUND_	FIC_INDEX_	Interaction
*H. pylori* 7189	CLR + **2b**	0.25	0.25	0.5	synergistic
	MTZ + **2b**	0.25	0.5	0.75	additive
	LEV + **2b**	0.25	0.5	0.75	additive
*E. faecalis* 37VRE	GM + **4b**	0.0625	0.5	0.5625	additive
	IMP + **4b**	1	1	2	neutral
	VA + **4b**	0.5	0.125	0.625	additive
	GM + **8b**	1	1	2	neutral
	IMP + **8b**	1	1	2	neutral
	VA + **8b**	0.125	0.25	0.375	synergistic
*S. aureus* KJ	GM + **4b**	0.125	0.25	0.375	synergistic
	E + **4b**	0.25	0.25	0.5	synergistic
	GM + **8b**	0.25	0.25	0.5	synergistic
	E + **8b**	0.25	0.25	0.5	synergistic
*K. pneumoniae* N111	GM + **6b**	1	1	2	neutral
	IMP + **6b**	1	1	2	neutral
	CPL + **6b**	1	1	2	neutral
*A. baumannii* 2800	GM + **3b**	1	1	2	neutral
	IMP + **3b**	1	1	2	neutral
	CPL + **3b**	0.5	0.0625	0.5625	additive

CLR (clarithromycin), MTZ (metronidazole), LEV (levofloxacin), GM (gentamicin), IMP (imipenem), VA (vancomycin), E (erythromycin), CPL (colistin).

## Supplementary Materials

The supplementary files are available online. Table S1. Concentrations of antibiotics and naringenin derivatives/their oximes combinations tested in the checkerboard assay; Figure S1. ^1^H-NMR (600 MHz, Chloroform-*d*) spectrum of 7-*O*-hexylnaringenin (**8a**); Figure S2. ^13^C-NMR (150 MHz, Chloroform-*d*) spectrum of 7-*O*-hexylnaringenin (**8a**); Figure S3. ^1^H-NMR (600 MHz, Chloroform-*d*) spectrum of 7,4′-di-*O*-hexylnaringenin (**9a**); Figure S4. ^13^C-NMR (150 MHz, Chloroform-*d*) spectrum of 7,4′-di-*O*-hexylnaringenin (**9a**); Figure S5. ^1^H-NMR (600 MHz, Acetone-*d_6_*) spectrum of 7-*O*-hexylnaringenin oxime (**8b**); Figure S6. ^13^C-NMR (150 MHz, Acetone-*d_6_*) spectrum of 7-*O*-hexylnaringenin oxime (**8b**); Figure S7. ^1^H-NMR (600 MHz, Acetone-*d_6_*) spectrum of 7,4′-di-*O*-hexylnaringenin oxime (**9b**); Figure S8. ^13^C-NMR (150 MHz, Acetone-*d_6_*) spectrum of 7,4′-di-*O*-hexylnaringenin oxime (**9b**).
