# Supplementary material for: Antimicrobial O-Alkyl Derivatives of Naringenin and Their Oximes Against Multidrug-Resistant Bacteria
Source: Molecules. 2020 Aug 10;25(16):3642. doi: 10.3390/molecules25163642 (PMC7464300; doi:10.3390/molecules25163642)
Supplement: Supplementary file 1 [file molecules-25-03642-s001.pdf]

Antimicrobial *O*-Alkyl Derivatives of Naringenin and Their Oximes against Multidrug-Resistant Bacteria

Anna Duda-Madej [1], Joanna Kozłowska [2,*], Paweł Krzyżek [1], Mirosław Anioł [2], Alicja Seniuk [1], Katarzyna Jermakow [1] and Ewa Dworniczek [1,*]

[1] Department of Microbiology, Faculty of Medicine, Wroclaw Medical University, Chałubińskiego 4, 50-368 Wrocław, Poland; anna.duda-madej@umed.wroc.pl (A.D.-M.); krojcerpawel@gmail.com (P.K.); alicja.seniuk@umed.wroc.pl (A.S.) katarzyna.jermakow@umed.wroc.pl (K.J.)
[2] Department of Chemistry, Faculty of Biotechnology and Food Science, Wrocław University of Environmental and Life Sciences, Norwida 25, 50-375 Wrocław, Poland; miroslaw.aniol@upwr.edu.pl
* Correspondence: joannakozlowska3@gmail.com (J.K.); ewa.dworniczek@umed.wroc.pl (E.D.)

Academic Editor: Diego Muñoz-Torrero
Received: 13 July 2020; Accepted: 8 August 2020; Published: 10 August 2020

Abstract: New antimicrobial agents are needed to address infections caused by multidrug-resistant bacteria. Here, we are reporting novel *O*-alkyl derivatives of naringenin and their oximes, including novel compounds with a naringenin core and *O*-hexyl chains, showing activity against clinical strains of clarithromycin-resistant *Helicobacter pylori*, vancomycin-resistant *Enterococcus faecalis*, methicillin-resistant *Staphylococcus aureus*, and beta-lactam-resistant *Acinetobacter baumannii* and *Klebsiella pneumoniae.* The minimum inhibitory concentrations (MICs), which provide a quantitative measure of antimicrobial activity, were in the low microgram range for the selected compounds. Checkerboard assays for the most active compounds in combination with antibiotics revealed interactions that varied from synergistic to neutral.

Keywords: naringenin; *O*-alkyl derivative of naringenin; oxime; multidrug-resistant pathogens

Table S1. Concentrations of antibiotics and naringenin derivatives/their oximes combinations tested in the checkerboard assay.

Figure S1. ^{1}H-NMR (600 MHz, Chloroform-*d*) spectrum of 7-*O*-hexylnaringenin (**8a**)

Figure S2. ^{13}C-NMR (150 MHz, Chloroform-*d*) spectrum of 7-*O*-hexylnaringenin (**8a**)

Figure S3. ^{1}H-NMR (600 MHz, Chloroform-*d*) spectrum of 7,4′-di-*O*-hexylnaringenin (**9a**)

Figure S4. ^{13}C-NMR (150 MHz, Chloroform-*d*) spectrum of 7,4′-di-*O*-hexylnaringenin (**9a**)

Figure S5. ^{1}H-NMR (600 MHz, Acetone-d_6) spectrum of 7-*O*-hexylnaringenin oxime (**8b**)

Figure S6. ^{13}C-NMR (150 MHz, Acetone-d_6) spectrum of 7-*O*-hexylnaringenin (**8b**)

Figure S7. ^{1}H-NMR (600 MHz, Acetone-d_6) spectrum of 7,4′-di-*O*-hexylnaringenin oxime (**9b**)

Figure S8. ^{13}C-NMR (150 MHz, Acetone-d_6) spectrum of 7,4′-di-*O*-hexylnaringenin (**9b**)

	Strains	Combination of antibiotics and compounds	Concentrations of tested antibiotics [µg/mL]	Concentrations of tested *O*-alkyl naringenin derivatives and their oximes [µg/mL]
1	*Helicobacter pylori* 7189	Clarithromycin + **2b**	0.25 – 128	1 – 16
		Levofloksacin + **2b**	0.015 – 8	1 – 16
		Metronidazole + **2b**	0.125 – 64	1 – 16
2	*Enterococcus faecalis* 37VRE	Gentamicin + **4b**	1 – 1024	1 – 64
		Imipenem + **4b**	0.0078 – 8	1 – 64
		Vancomycin+ **4b**	0.5 – 512	1 – 64
		Gentamicin + **8b**	1– 1024	0.125 – 8
		Imipenem + **8b**	0.07 – 8	0.125 – 8
		Vancomycin + **8b**	0.5 – 512	0.125 – 8
3	*Staphylococcus aureus* MRSA KJ	Gentamicin + **4b**	1 – 1024	0.5 – 32
		Erythromycin + **4b**	1 – 1024	0.5 – 32
		Gentamicin + **8b**	1 – 1024	0.125 – 8
		Erythromycin + **8b**	1 – 1024	0.125 – 8
4	*Klebsiella pneumoniae* N111	Colistin + **6b**	0.0019 – 2	8 – 512
		Gentamicin + **6b**	0.015 – 16	8 – 512
		Imipenem + **8b**	0.0019 – 2	8 – 512
5	*Acinetobacter baumannii* 2800	Colistin + **3b**	0.0039 – 4	8 – 512
		Imipenem + **3b**	0.0612– 64	8 – 512
		Gentamicin + **3b**	1 – 1024	8 – 512

Table S1. Concentrations of antibiotics and naringenin derivatives/their oximes combinations tested in the checkerboard assay.

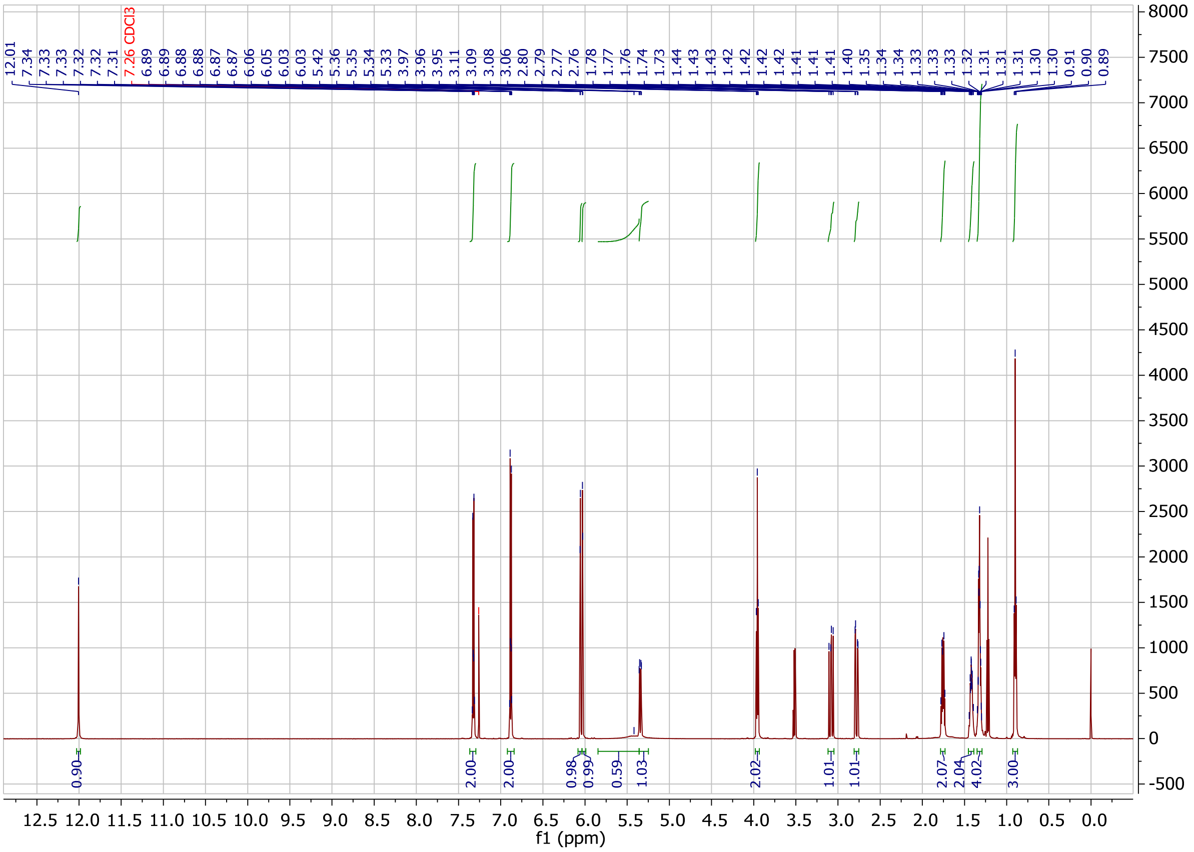

Figure S1. ^{1}H-NMR (600 MHz, Chloroform-*d*) spectrum of 7-*O*-hexylnaringenin (**8a**)

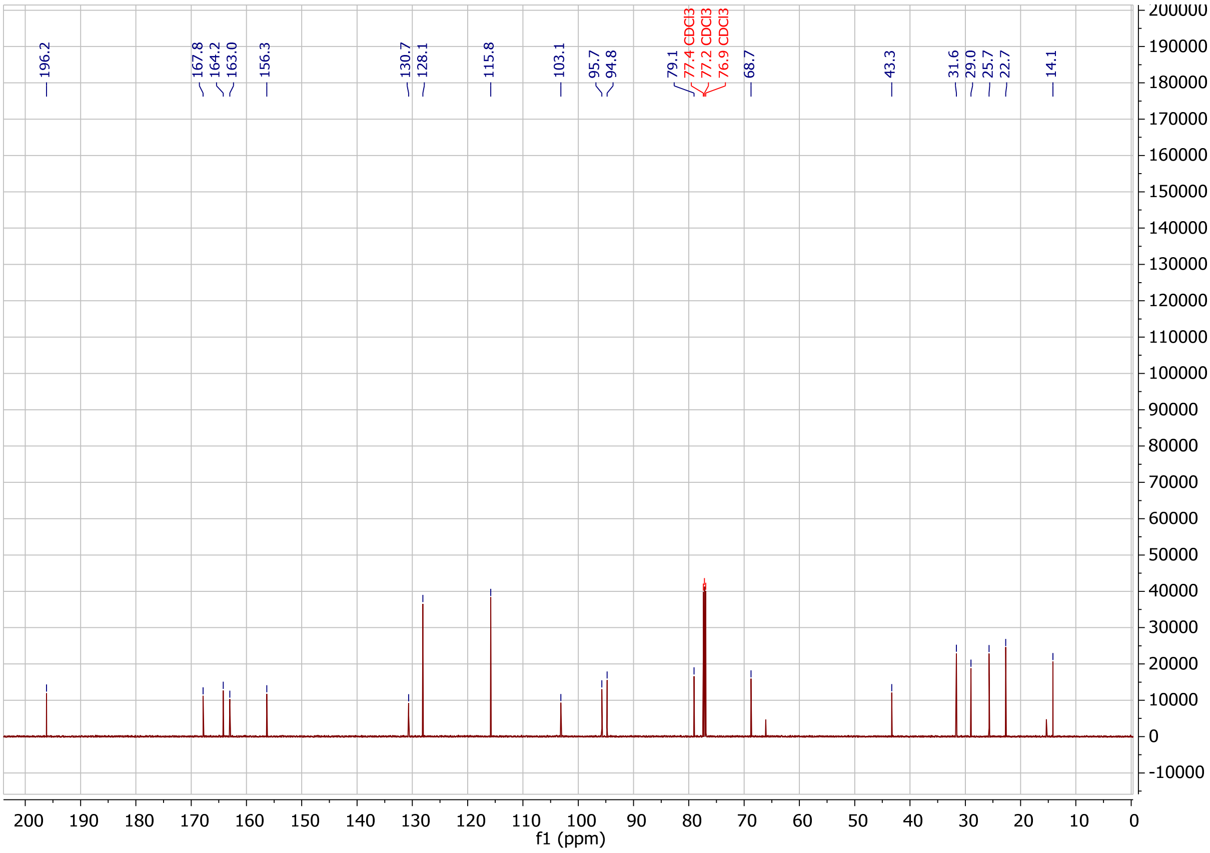

Figure S2. ^{13}C-NMR (150 MHz, Chloroform-*d*) spectrum of 7-*O*-hexylnaringenin (**8a**)

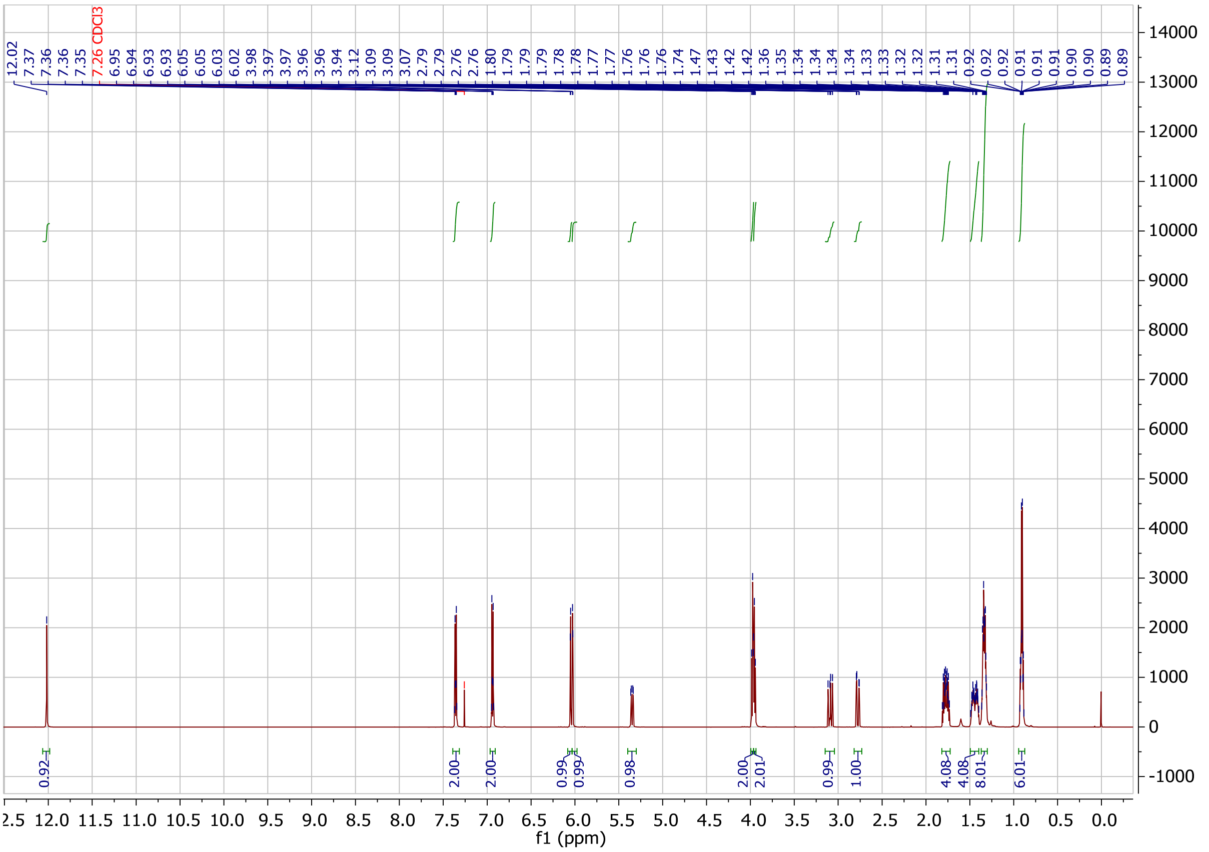

Figure S3. ^{1}H-NMR (600 MHz, Chloroform-*d*) spectrum of 7,4′-di-*O*-hexylnaringenin (**9a**)

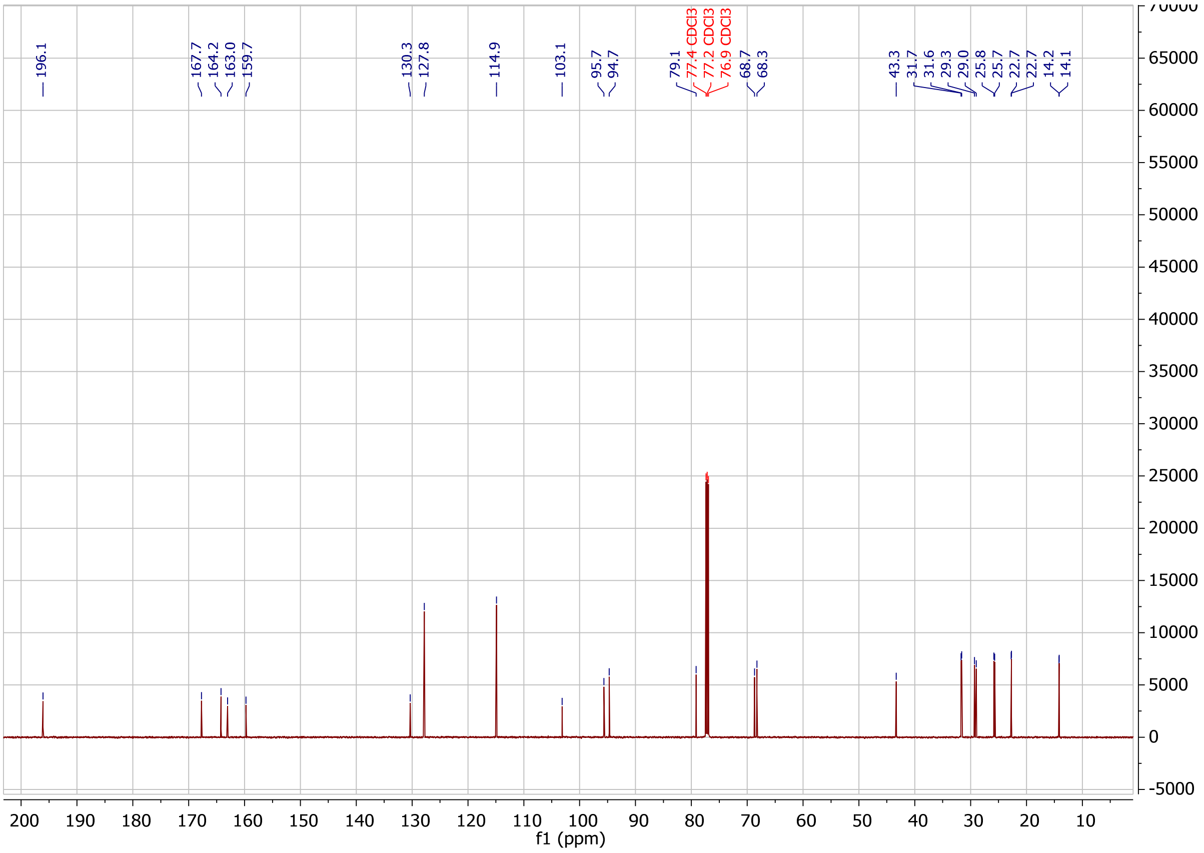

Figure S4. ^{13}C-NMR (150 MHz, Chloroform-*d*) spectrum of 7,4′-di-*O*-hexylnaringenin (**9a**)

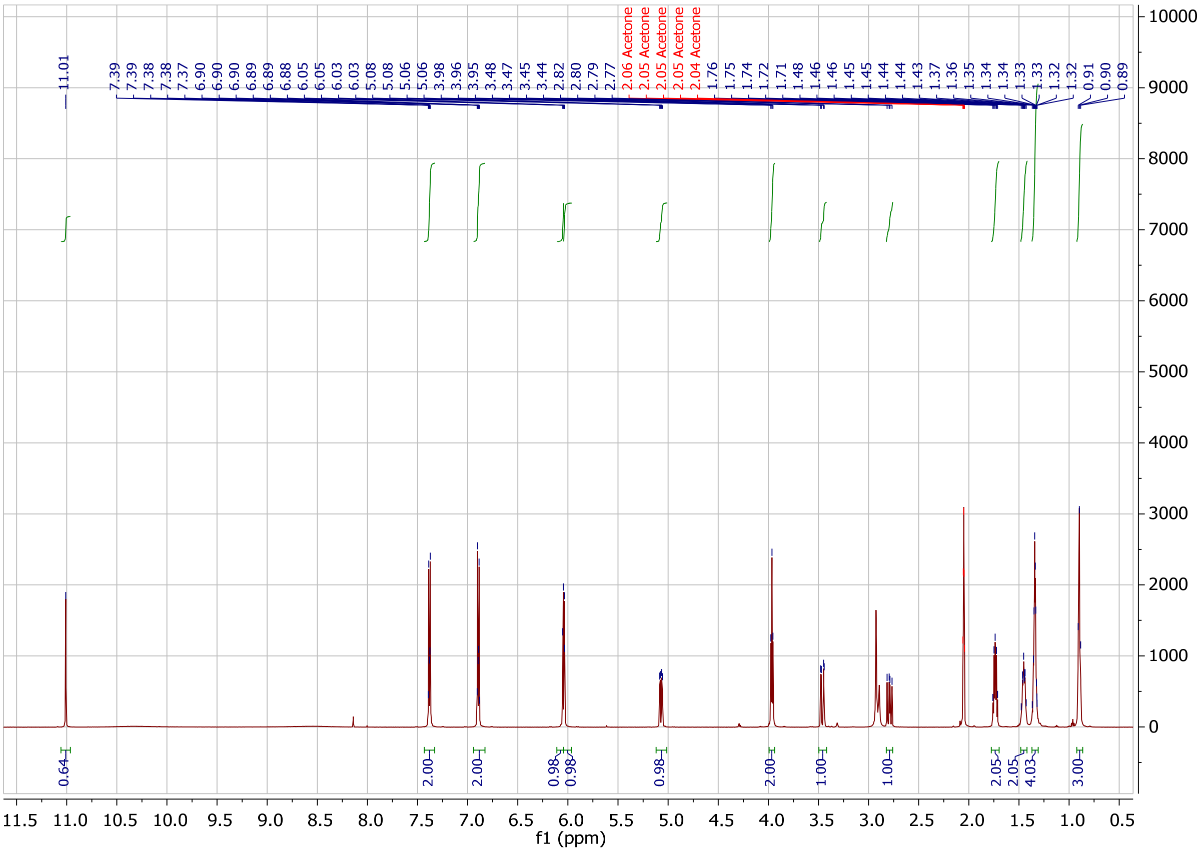

Figure S5. ^{1}H-NMR (600 MHz, Acetone-*d*$_6$) spectrum of 7-*O*-hexylnaringenin oxime (**8b**)

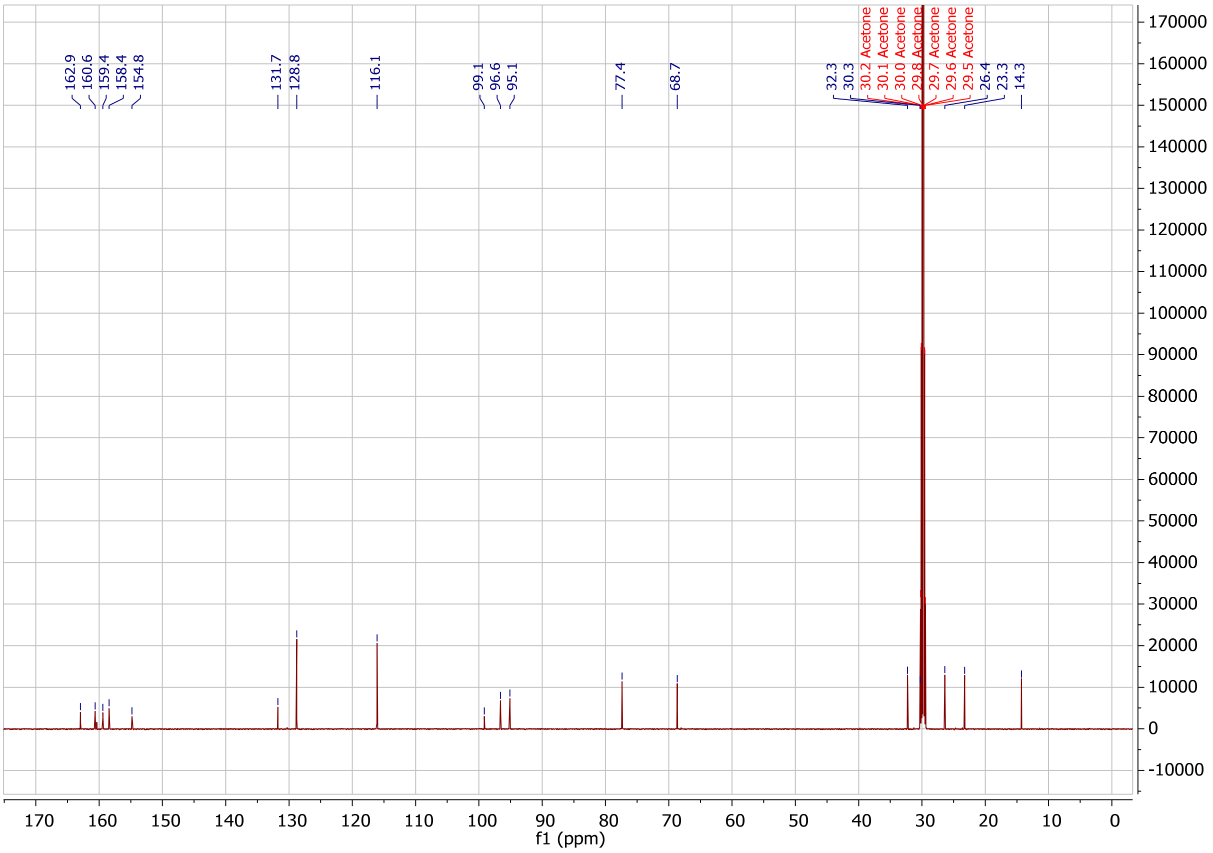

Figure S6. ^{13}C-NMR (150 MHz, Acetone-d_6) spectrum of 7-*O*-hexylnaringenin oxime (**8b**)

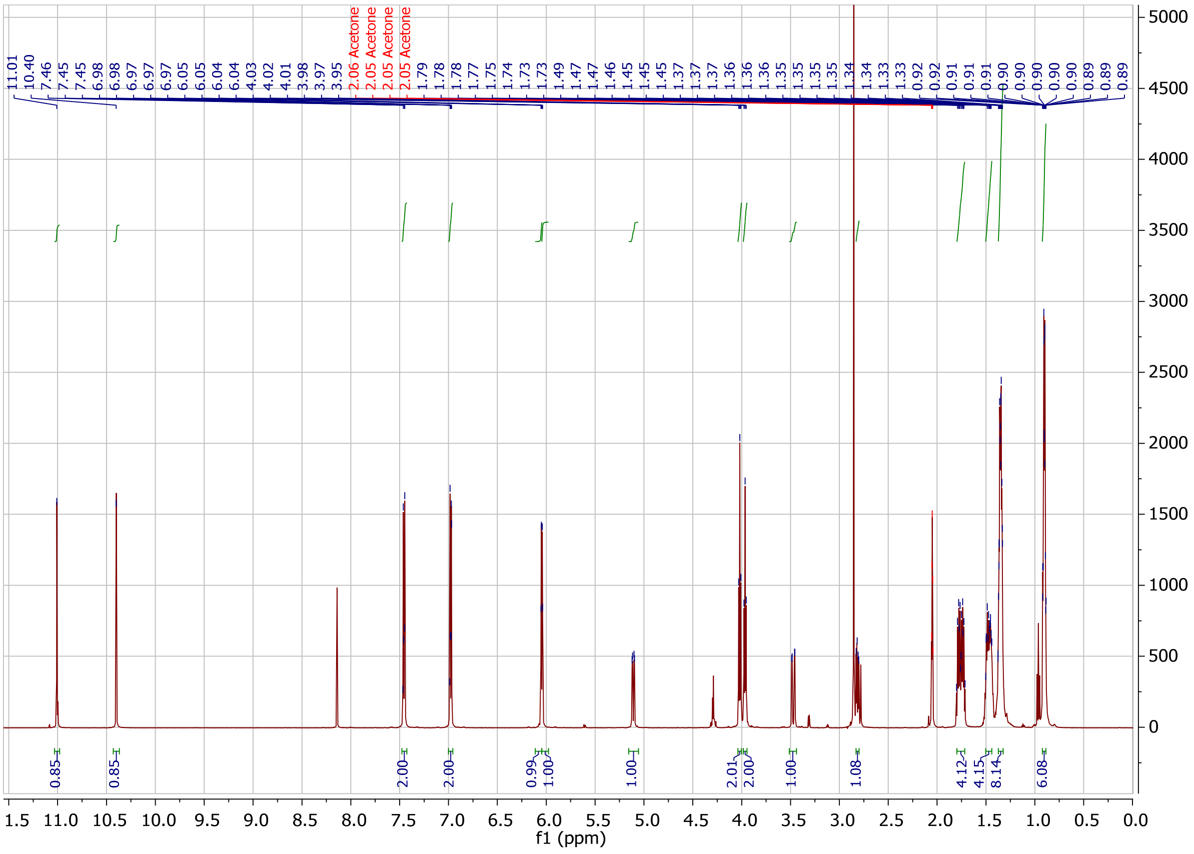

Figure S7. ^{1}H-NMR (600 MHz, Acetone-d_6) spectrum of 7,4′-di-*O*-hexylnaringenin oxime (**9b**)

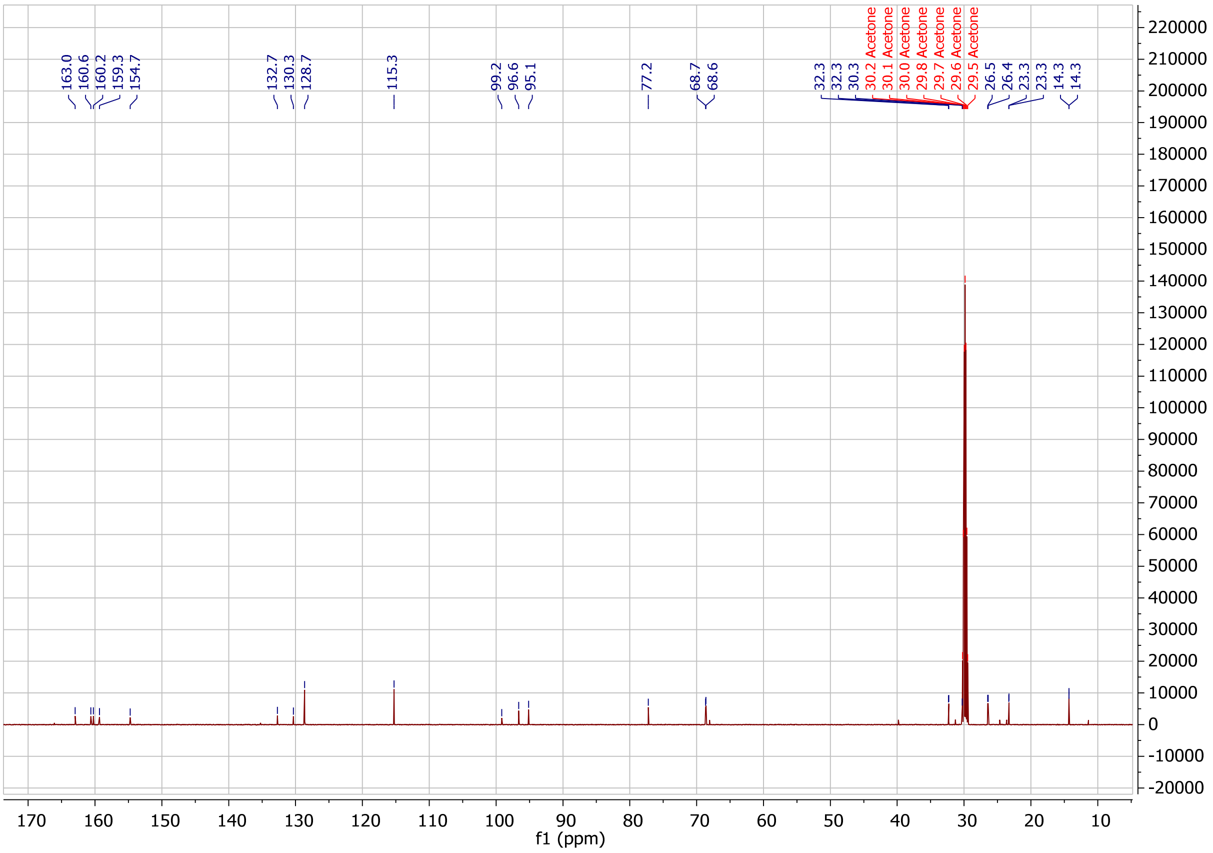

Figure S8. ^{13}C-NMR (150 MHz, Acetone-d_6) spectrum of 7,4′-di-*O*-hexylnaringenin oxime (**9b**)
